# Mechanosignals in abdominal aortic aneurysms

**DOI:** 10.3389/fcvm.2022.1021934

**Published:** 2023-01-09

**Authors:** Christiana Lowis, Aurellia Ramara Winaya, Puja Kumari, Cristobal F. Rivera, John Vlahos, Rio Hermantara, Muhammad Yogi Pratama, Bhama Ramkhelawon

**Affiliations:** ^1^Division of Vascular and Endovascular Surgery, Department of Surgery, New York University Langone Medical Center, New York, NY, United States; ^2^Department of Biomedicine, Indonesia International Institute for Life-Sciences, Jakarta, Indonesia; ^3^Department of Cell Biology, New York University Langone Medical Center, New York, NY, United States

**Keywords:** abdominal aortic aneurysm, mechanosignals, shear stress, mechanotransduction, mechanical stress, vascular pathology

## Abstract

Cumulative evidence has shown that mechanical and frictional forces exert distinct effects in the multi-cellular aortic layers and play a significant role in the development of abdominal aortic aneurysms (AAA). These mechanical cues collectively trigger signaling cascades relying on mechanosensory cellular hubs that regulate vascular remodeling programs leading to the exaggerated degradation of the extracellular matrix (ECM), culminating in lethal aortic rupture. In this review, we provide an update and summarize the current understanding of the mechanotransduction networks in different cell types during AAA development. We focus on different mechanosensors and stressors that accumulate in the AAA sac and the mechanotransduction cascades that contribute to inflammation, oxidative stress, remodeling, and ECM degradation. We provide perspectives on manipulating this mechano-machinery as a new direction for future research in AAA.

## 1. Divergent mechanosignals during AAA development

Although the advancement of surgical approaches as curative management of abdominal aortic aneurysm (AAA) has become more sophisticated in recent years, there is still a shortage of non-interventional treatments available to curb the growth of aneurysm sacs, thereby reducing the risk of life-threatening aortic rupture (1). This limitation is mainly due to the gap in knowledge regarding complex pathological signaling networks that are present during different phases of AAA. Clinical studies have established that the aorta is hemodynamically altered during the early and progressing phases of AAA, which is associated with sac expansion and rupture (2–4). However, the exact mechanobiology during each specific stage of the disease remains uncertain. Understanding how the aortic cells can sense these hemodynamic changes and the mechanisms they employ to convert the mechanical stimuli into biochemical signals that modulate AAA initiation, progression and rupture are essential to design targetable therapy, consolidating prediction markers and, most importantly, improving our understanding of the complex pathobiology of AAA.

**FIGURE 1:**
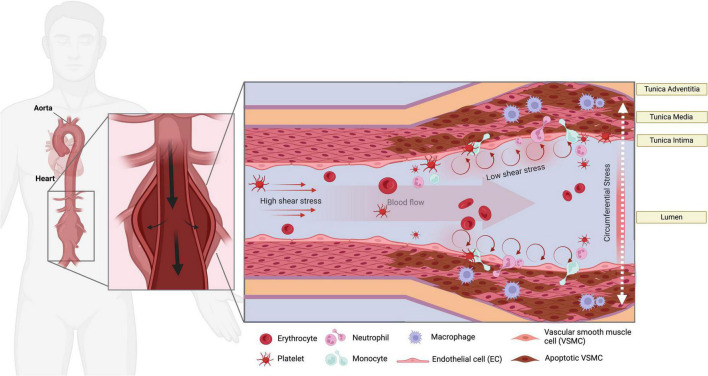
Mechanical stressors in AAA. The endothelial layer of the aorta is subjected to high shear stress (HSS) in linear segments of the aorta. Turbulent low shear stress (LSS), along with the increase of circumferential stress imposed by blood pressure and aortic stiffness, are sensed at curved areas. LSS can over-activate the ECs to attract immune cells and contribute to the pathological remodeling and SMCs apoptosis *via* mechanosensory machinery in the ECs. Platelet activation and accumulation in the AAA sac likely occur during the progression of AAA.

Within the vascular wall, the cells and composite extracellular matrix (ECM) are constantly exposed to the toned physical forces and mechanical stimuli from the luminal and adventitial microenvironment (5). Endothelial cells (ECs), vascular smooth muscle cells (SMCs), inflammatory cells residing in the aortic tissue, and critical elements of the ECM (collagen, elastin, and proteoglycans) are subjected to cyclic stretch, circumferential stress, and shear stress due to changes in flow and pressure in the vasculature (6–8). These applied forces could be extended beyond the endothelium layer and result in circumferential stress and stretch in the tunica media and adventitia, which likely contribute to the initial steps that promote AAA onset (9). ECs, SMCs, fibroblasts, and immune cell infiltrates likely sense these tensions, thereby triggering several signaling pathways that dictate their function, a mechanism known as mechanotransduction (10, 11). From the mechanical point of view, the critical state of AAA is formed when the mechanical stresses (internal forces per unit area) within the aneurysmal wall exceed the ability of the wall to withstand these stresses (Figure 1). The circumferential stress imposed by blood pressure, along with increased perivascular constraints and microstructural changes, are strongly associated with the decline of the overall wall strength and likely lead to rupture (12). To date, the current literature demonstrates that these signals could mediate vascular remodeling, immune cell activation, reactive oxygen species (ROS) generation, and apoptosis in AAA.

## 2. Endothelial and smooth muscle cell mechanosensors in AAA

The vascular ECs, which line the inner surface of blood vessels in direct contact with blood flow, are strategically positioned to sense and respond to these hemodynamic and biochemical changes, thereby tuning the vascular tone (13–16). In physiological states, the endothelial layer of linear segments of the aorta is subjected to high shear stress (HSS), a frictional laminar force (15–20 dynes/cm^2^) generated by blood flow, which regulates local oxidative stress, reinforces the inflammatory barrier capacity and protects the overall segment against pathological remodeling (17, 18). In curved areas of the aortic tree, pulsatile blood flow with turbulent patterns produce a range of low shear stress (LSS) magnitudes (0–2 dynes/cm^2^), which over-activate the ECs, thereby reprogramming their function to create oxidative stress and attract immune cells circulating in the blood (19, 20). HSS and LSS are inherent to the nature of the architecture of the aorta comprised of linear segments, curvatures, and bifurcations. The abdominal portion of the aorta is an intriguing hemodynamic carrefour comprised of several projections of the renal, mesenteric, and gonadal arteries that generate a spectrum of microflow LSS patterns at the inner curvatures of the bifurcations. ECs are equipped with multiple sophisticated mechanosensory machineries present at their membrane, including ion channels (21–24), tyrosine kinase receptors (25, 26), G protein-coupled receptors (GPCR) (27), integrins (28, 29), and the cytoskeleton (30, 31). Boyd et al. (2) demonstrated the presence of altered shear stress in patients with AAA, which was significantly upregulated at sites of AAA rupture. The cytoprotective ECs phenotype was shown to be generated under HSS during physiological conditions (8, 17). *In vivo* studies by Xie et al. (32) showed that ECs subjected to LSS and disturbed flow undergo increased apoptosis through increased monocyte adhesion and inflammatory responses. While HSS is physiologically observed in the normal abdominal aorta, the laminar flow pattern is switched to LSS patterns exerting additional stress on the endothelium once the aortic tissue is deformed into a bulged structure, introducing new curvatures within the AAA sac (33). Observations in patients with aneurysms have demonstrated that LSS magnitude is co-incident with rupture sites (2–4, 12, 34), further emphasizing the association between LSS and the severity of AAA. While these studies suggest that LSS could be an important triggering factor that elicits rupture, it might also reflect a consequence of altered aortic microarchitecture at the rupture site. Notably, other mechanical parameters were not assessed in these studies, likely due to the complexity of capturing timed rupture events, which would have provided a comprehensive pattern of hemodynamic forces that manifest during rupture. Furthermore, it is also likely that LSS leads to the activation of the endothelium at the initial phases of AAA formation, coinciding with the deformation of the aortic tissue from a luminal tube into a bulged structure. However, these studies are difficult to perform in human studies as they require close monitoring of patients at risk to capture the hemodynamics at these germinal stages of AAA formation.

The altered aortic biomechanics at critical stages during the onset, progression, and rupture of AAA likely incite downstream multifactorial mechanotransduction signaling patterns that intersect with pathological vascular remodeling programs in ECs. Notably, whole-genome sequencing performed by Erhart et al. (35) showed the association between ECM genes with different shear stress profiles in human aneurysmal tissue. In patients with LSS, their genetic profile significantly correlated with the upregulation of Laminin subunit alpha-4 (LAMA4) and Sushi-repeat containing protein x-linked-2 (SRPX2) gene that provoked ECM degradation and the downregulation of several pro-inflammatory chemokines that suppress inflammation, suggesting that shear stress dynamics are capable of directly modulating ECM degradation programs through distinct transduction pathways during the progression of AAA. Moreover, several elastin-derived peptides and enzymes generated by the degradation of elastin fibers have been linked to elicit a pro-inflammatory environment and further drive elastin degradation during AAA progression (6, 36), even though their precise role in the mechanosignaling pathways are yet to be elucidated.

### 2.1. Ion channel mechanotransduction pathways in AAA

**FIGURE 2:**
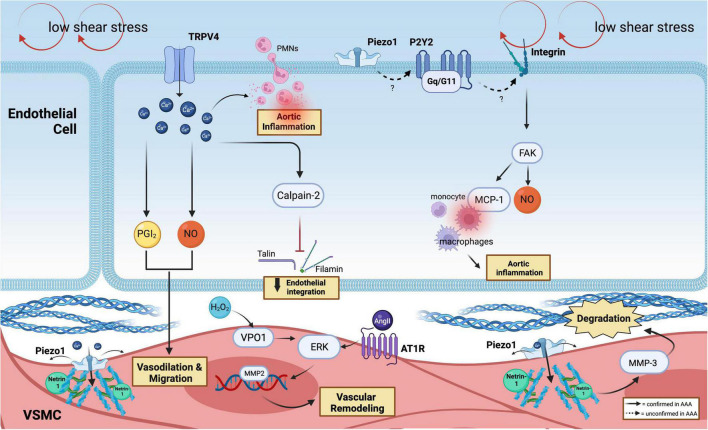
Mechanoreceptors involved in AAA. In the ECs, low shear stress is sensed by the ion channel (TRPV4 and Piezo1) and purinergic-coupled receptors (P2Y2-Gq11). Through Ca^2+^-responses, which produce PGI_2_ and NO, SMCs vasodilate and migrate, followed by the release of calpain-2 that induce the loss of endothelial integration. Ca^2+^ flux facilitates PMNs infiltration and inflammation. P2Y2-Gq11 is predicted to activate integrin, which can drive FAK activation. FAK plays a vital role in mediating and recruitment of macrophages *via* MCP-1 and participating in the production of NO. Netrin-1 promotes Piezo1 opening in SMCs, which activates MMP3 and causes collagen degradation. PECAM-1, VE-cadherin, and VEGFR2 can further activate FAK and MCP-1 secretion that recruits macrophages. The phenotypic switching of SMCs is induced by the H_2_O_2/_VPO1/ERK pathway in AAA and *via* AT1R activation.

It has been widely recognized that ion channels can function as essential mechanotransducers that maintain the dynamic balance in the pulsatile vascular wall. The transient receptor potential isoform-4 (TRPV4) is a calcium-permeable channel commonly expressed by ECs and SMCs, which can regulate calcium influx *via* shear stress sensing and subsequently induce vasodilation (NO and PGI2) in AAA when shear stress increases (37–41). Calcium-induced membrane depolarization is considered a major event that occurs following the activation of the ion channel, which potentially contributes to the early development of AAA. Shannon et al. (42) demonstrated that a specific TRPV4 antagonist, GSK2193874, was able to attenuate aortic growth and decrease pro-inflammatory cytokines in both angiotensin II (AngII)-induced AAA in ApoE^–/–^ mice and in ECs in culture, thereby reducing the activation of SMCs and trans-endothelial migration during AAA formation (Table 1 and Figure 2).

**TABLE 1:** Summary of mechanosensors, actions, and their phenotypic effects in AAA.

Cells	Mechanosensors	Actions	Phenotypic effects	References
SMCs	Piezo1	Increase in cytoskeleton cross-linking of α-actinin2 regulated by Netrin-1, elevated MMP3	Matrix stiffness alters SMCs function, ECM degradation	(52)
ECs and SMCs	TRPV4	Release of vasodilators mediators (NO, PGI2) through Ca^2+^ influx. Increased migration of myeloid cells, elevated MMP2, and MMP9	Vasodilation and migration of SMCs, aortic inflammation, vascular remodeling	(42)
ECs	Integrin α5β1	FAK activation leading to NO production	Vasodilation and migration of SMCs	(65)
SMCs	AT1R	Activate ERK1/2 signaling	ECM degradation, SMCs migration, release of proinflammatory cytokines	(69)

Several *in vivo* studies have been conducted in rodents with AAA modeled by calcium chloride (CaCl_2_) or calcium phosphate (CaPO_4_) treatment. These models are based on vasodilation, calcification, oxidative stress, and SMCs apoptosis (43, 44) that culminate in ECM degradation and AAA onset. This suggests the critical role of calcium-induced mechanisms as central regulatory hubs in the pathogenesis of AAA. Indeed, Ca^2+^ is a vital cofactor that fuels the activation of matrix metalloproteinases (MMPs), widely regarded as the pathological culprits that exaggeratedly degrade the ECM during AAA. Hence, strategies to manipulate Ca^2+^ levels are desirable to curb AAA. However, it is worth noting that the use of broad-spectrum Ca^2+^ blockers did not show beneficial effects in treating thoracic aneurysm progression associated with Marfan syndrome (45). Future studies are needed to delineate the triggers and downstream signaling pathways that interconnect with Ca^2+^ signaling in distinct vascular cell types during AAA to optimally devise strategies to specifically modulate pathological Ca^2+^ programs such as those mediated by Piezo1, as discussed later.

Li et al. (46) showed that the activation of calpain, calcium-dependent cysteine proteases, through shear stress-coupling could target the ECM cytoskeleton. Specifically, calpain-2 could fragment talin and filamin, disrupting endothelial organization and alignment that facilitates ECs and SMCs migration in AngII-induced AAA in LDLR^–/–^ mice (47). Subramanian et al. (48) later conducted *in vivo* pharmacological inhibition of a novel calpain inhibitor, BDA-410, which successfully reduced the incidence and development of AAA by attenuating the activation of MMP12, pro-inflammatory cytokines, and macrophage infiltration into the aorta using similar AAA mice models.

The recently discovered ion channel mechanosensor, Piezo1, has gained much interest in the cardiovascular field. Piezo1 channel exhibits a three-bladed, propeller-shaped homotrimer structure with a dome mechanism, which sustains a potential energy source for its mechanosensitive gating (49). Accordingly, Piezo1 is known to be activated with a half-maximal shear stress intensity of 57 ± 3 dynes/cm^2^ that leads to a cascade of downstream signaling pathways in ECs and SMCs (14). Indeed, Kang et al. (24) have shown the alteration of both physiological and functional properties of ECs following shear-induced activation of Piezo1. In atherosclerosis, Albarrán-Juárez et al. (50) demonstrated the role of Piezo1 as a shear stress sensor, thereby causing the polarization of ECs under disturbed flow, triggering the release of ATP that activates the P2Y2-Gq/G11 coupled-receptor in promoting integrin activation. This could be a relevant pathway to be examined in AAA, given the similar shear perturbations in the aneurysmal sac. Further investigations are warranted using the inducible conditional loss of Piezo1 in ECs since Piezo1 disruption during early embryogenesis has led to lethal vascular impairment in mice (46, 51).

The discovery made by Qian et al. (52) remains the only study that directly addressed the role of Piezo1 in AAA. Unlike ECs, SMCs are predominantly activated *via* mechanical stretch that influences the structural organization and signaling in SMCs (53, 54). Arterial stiffness and increased cytoskeleton cross-linking of α-actinin2 by Netrin-1 in SMCs were observed in AAA walls, powering the opening of Piezo1 (52) and leading to downstream activation of MMP3. Thus, we showed the detailed signaling trajectory that lead to the activation of Piezo1 in AAA, which was further supported by atomic force microscopy (AFM) analysis, which demonstrated elevated stiffness within the SMCs. In addition, single-cell RNA sequencing of mice AAA specimens revealed the increase of Piezo1 expression in SMCs in AAA groups, suggesting the transcriptional regulation of Piezo1.

Currently, the GsMTx4 peptide is the only Piezo1 inhibitor tested in AAA, which has been shown to repress matrix degradation *via* MMP3 and reduce aortic dilatation in the AngII and elastase-induced AAA mice models (52). However, GsMTx4 was reported to exert off-target effects, such as the inhibition of voltage-gated sodium channels (55), warranting further studies to design more specific inhibitors. Hadi et al. (56) described that the deficiency of MMP3 in mice protects against AAA, suggesting that this ECM degrading program could be at the intersection of mechanosensory signals during AAA development.

### 2.2. Integrin-mediated pathways in AAA

In static conditions, the mechanosensitive integrins are inactively assembled as dormant unphosphorylated complexes (57). Shear stress patterns can activate signaling *via* several integrins such as PECAM-1, vascular endothelial cadherin (VE-cadherin), and vascular endothelial growth factor-2 (VEGFR2), through conformational changes and specific interactions of the α and β subunits which can drive focal adhesion kinase (FAK) activation (58–60). FAK plays an essential role in macrophage-mediated chronic progression of AAA as FAK inhibitor attenuates macrophage inflammatory responses during AAA development (61). According to prior reports, FAK can stimulate fibroblasts to secrete monocyte chemoattractant protein (MCP-1), which then causes macrophage recruitment. MCP-1 is essential for the onset of AAA and vascular inflammation (62).

It was previously reported that the integrin subset, α5β1, selectively binds collagen and fibronectin (63). Cheuk and Cheng showed that the distinct reduction in integrin α5β1 expression was seen in human aneurysmal aortic tissues and was associated with a reduction in the density of SMCs (64). Therefore, the lack of this integrin subset may impede matrix protein attachment and alter the geometry of the aortic media, potentially forming aneurysms. This discovery is in line with other studies which postulated that the absence of β1 may influence vascular mechano-adaptivity and alter its phenotype (12, 65). The activation of β1 integrin by shear stress using the fibronectin–integrin–cytoskeleton connection in primary human umbilical vein ECs (HUVEC) subjected to different levels of shear stress confirmed these studies. It was found that β1 integrin serves as a signaling mediator of three different levels of shear stress (0.04, 2.0, and 3.7 Pa), suggesting the critical role of this protein in scaling the mechanosensory potential of ECs (66).

### 2.3. G-protein coupled receptors (GPCRs) signaling pathway in AAA

G-Protein coupled receptors are characterized by the presence of seven transmembrane alpha-helics and are considered the largest protein superfamily observed in a higher organism (67). Several GPCRs, such as β_2_ARs (β_2_-adrenergic receptor) and AT1R (angiotensin II type I receptor), are present in ECs and SMCs, regulating an array of functions including vascular tone, angiogenesis, and cell proliferation. AT1R has also been demonstrated to act as a mechanosensor in ECs and can be activated under excess mechanical stretch (68). It was observed that the mechanical activation of AT1R in hypertensive mice amplifies AAA growth and significantly elevates the activity of ERK1/2 in hypertensive (BPH/2) and normotensive (BPH/3) mice (69). The hyperactivation of AT1R may increase macrophage infiltration, which leads to the production of inflammatory cytokines, ECM degradation, and sac expansion, with a propensity to rupture (70, 71). Despite the need for mechanistic validation study in larger animal models, AT1R blockers, such as Losartan and Telmisartan (69, 72), were shown to slow AAA growth and rupture in elastase-treated brown rats and hypertensive BPH/2 mice even though conflicting result was observed from a randomized clinical trial in patients with AAA (73).

## 3. Mechanical stress, inflammation, and redox stress circuits in AAA

AAA is established as an inflammatory and redox condition of the aortic tissue. Various immune cells interact with each other and mediate crosstalk with vascular cells within the AAA sac. To this end, persistent LSS that fuel the release of inflammatory cytokines may encourage matrix remodeling and severe inflammation influencing aneurysmal growth (74). It is established that LSS is the prime origin of elevated levels of reactive oxygen species (ROS) and inflammatory genes that sustain ECM degradation in AAA through nuclear factor kappa B (NF-κB), MAP protein kinase (MAPK), and transforming growth factor beta (TGF-β) pathways (75–77).

Oxidative stress plays a significant role in mechanotransduction (78, 79). ROS persists at high levels in response to low and turbulent SS. Flow-mediated ROS such as O_2_^–^ may transform into H_2_O_2_, and through the Fenton reaction, H_2_O_2_ can spontaneously transform into hydroxyl radical (OH), all of which have been detected in increased amounts in AAA samples and positively associated with aneurysm size and mortality risk of patients (80). There are multiple sources of ROS production such as uncoupled eNOS, xanthine oxidase (XO), cyclooxygenase, mitochondria, and NADPH oxidase (81). Shear stress may increase endothelial XO expression and activity by utilizing molecular oxygen as an electron acceptor together with H_2_O, O_2_^–^, and xanthine/hypoxanthine, XO produces O_2_^–^ and H_2_O_2_ (82). Mitochondrial ROS produced from aortic macrophages was also described to induce matrix degradation *via* receptor-interacting serine/threonine-protein kinase-3 (RIPK3) in AAA induced by injured lungs, suggesting the pathological role of RIPK3 to trigger ROS production (83). Moreover, it was observed that ROS produced from these pro-inflammatory macrophages can activate MMP12, subsequently leading to matrix degradation in the aorta and fueling AAA expansion (83). It is interesting to further explore whether this macrophage-derived ROS circuit is also induced by mechanical stresses with or without underlying inflammation. However, a clinical study performed in patients with AAA undergoing surgical repair showed that oxidase systems such as XO and mitochondria were not altered by their corresponding inhibitors in AAA (84). Mice with a deficiency in eNOS pre-uncoupled HPH-1 gene treated with angiotensin II spontaneously developed AAA and died from ruptured AAA. Oral administration of folic acid prevented AAA formation in these mice by restoring vascular remodeling through MMP2 and MMP9 reduced activity and alleviated macrophage accumulation in the wall (85).

**FIGURE 3:**
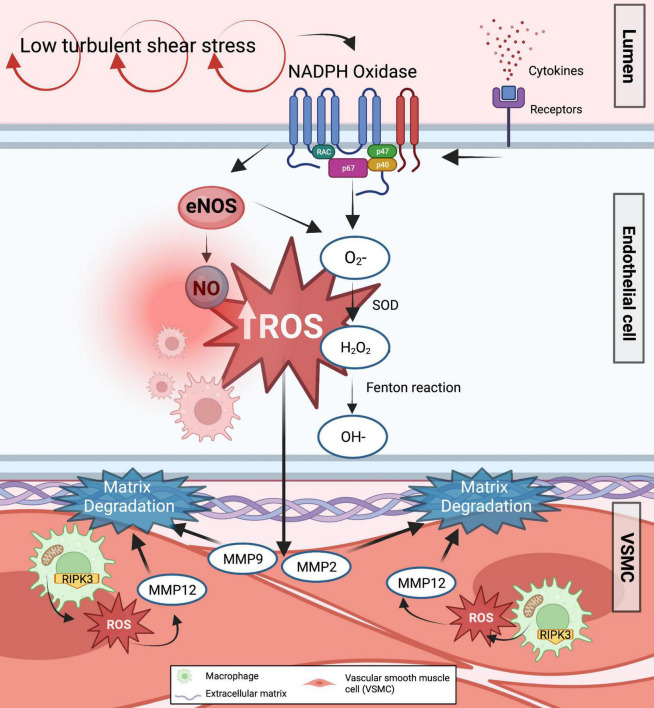
Formation of ROS in response to low shear stress in AAA. The majority of ROS primary sources, such as uncoupled eNOS and NADPH oxidase, are known to enhance ROS generation in response to low shear stress. As a result, various ROS are generated in the endothelium cells. Increased O_2_^–^ and H_2_O_2_ inactivates endothelium-derived NO synthase and takes up any NO produced while augmenting the transcriptional factors involved in the activation of downstream effectors related to matrix degradation and inflammation. Furthermore, mitochondrial ROS are produced from pro-inflammatory aortic macrophages *via* RIPK3, further inducing matrix degradation and AAA expansion.

NADPH oxidase is an important source of O_2_^–^ in AAA. Mechanical stimuli can trigger NADPH oxidase to utilize NADH/NADPH as an electron donor to reduce the molecular oxygen (86). In iNOS^–/–^ deficient mice, specific inhibition of NADPH oxidases successfully prevented aneurysm formation (87), suggesting a circuit between NADPH, iNOS, and NO levels in stimulating AAA progression (Figure 3).

## 4. Intraluminal thrombus and mechanosensing in AAA

In recent years, many studies have tried establishing the role of activated platelets in the development and progression of AAA. Aside from their crucial role in thrombosis and hemostasis, a non-occlusive intraluminal thrombus (ILT) formation is known to contribute to the pro-inflammatory and prothrombotic backdrop of AAA (88–90). It was initially hypothesized that ILT could act as a protective mechanism to reduce mechanical stress induced on the vascular wall (91). The formation of ILT occurs following platelet activation, aggregation, and adhesion in regions of LSS, subsequently decreasing the mechanical stress on the aortic wall and maintaining AAA stability (92). However, evidence shows that the presence of ILT is associated with the production of hypoxia, elastolysis, and pro-inflammatory microenvironment surrounding the aneurysmal wall (93). It was demonstrated that different shear stress patterns that might occur during thrombotic events within the aortic walls could contribute to the progression of ILT and consequently exacerbate AAA (94, 95). Based on these studies, it has been concluded that while there may be early beneficial effects of ILT, it is likely that the presence of ILT might have deleterious effects as the disease progresses.

Surprisingly, thrombus formation is also observed in regions exposed to low (13, 96) and oscillatory SS (97). High shear rates can further activate the von Willebrand factor (vwf), a key player in platelet adhesion (98). Whereas at LSS, fibrinogen binding to GPIIb/IIa is mainly responsible for aggregation (99). Previous clinical studies have shown the positive effect of anticoagulant therapy on lowering ILT thickness and volume (100). The exact mechanism of the role of platelet in the mechanotransduction is still unknown, whether the mechanical changes associated with AAA development subsequently modulate the phenotype of activated platelets, whether platelets contribute to the shear sensing of ECs and interfere with its mechanoreceptors within the AAA sac still warrants further investigation.

## 5. Mechanotransduction in AAA: A translational perspective

It is clear that mechanical disturbance in the aortic environment and the changes in mechanotransduction within aortic tissue are progressive events during AAA development; hence, monitoring these events in a clinical setting could be beneficial for improved surveillance and therapeutic purpose.

### 5.1. Tools to study mechanotransduction in AAA

Mimicking the cellular response to shear stress persists as the main challenge to study the mechanotransduction channels in AAA. Such studies require an appraisal of large animal or specific rodent models to observe vascular mechanical responsiveness while integrating several bioreactors and physical methods to apply flow to cells and specify the response of ECs from mass transport mechanisms (101). Bowden et al. (102) summarized the availability of *in vitro* and *in vivo* models to examine shear stress in AAA. Accordingly, porcine pancreatic elastase (PPE) infusion, along with partial ligation of the iliac artery in murine and rodent models, can increase mechanical stretch and shear stress and alter the morphology of AAA phenotypes (103, 104). However, the difference in SS experienced between murine and human vasculature can lead to significant variation and bias in translating the results effectively. The porcine model can be a helpful alternative due to comparable vascular anatomy and mechanical rheology with humans (102, 105). Induction of elastase along with β-aminopropionitrile (BAPN) in the porcine model was successfully created and generated an additional analogous AAA model sharing similar hallmarks with human AAA (106). Thus, these large animal models seem adequate to test novel therapies targeting mechanotransduction signaling pathways to curb AAA development.

In order to understand the mechanism by which cells respond to mechanical stress and the mechanotransduction signaling that drives AAA at the subcellular level, a stringent *in vitro* system is necessary to mimic *in vivo* conditions, which remain a challenge considering cells in culture are not subjected to constant stress, ECM microenvironment and physical forces observed *in vivo* (107). Several studies have tackled such issues by creating a multi-layered 3D-vascular scaffold that can mimic the biological vasculature as opposed to the traditional 2D culture of SMCs or ECs (108–110). Organ-on-chip platforms have been utilized to characterize the interactions between ECs and SMCs. Using a platform bioprinted into a microfluidic device, SMCs and ECs can be cocultured on a porous, tensile membrane that underwent mechanical stretch and shear stress, making this system suitable to delve into the mechanical mechanisms in AAA and for the testing of high-throughput drug candidates (110). Using this model, Chen et al. replicated the cyclic stretch of human pluripotent stem cell-derived aortic smooth muscle cells (hPSC-HASMCs) to study the effect of metformin in aortic aneurysm development. They identified that metformin is capable of targeting NOTCH1 signaling, thereby rescuing the SMCs pathological switch that occurs in AAA (111).

Bogunovic et al. also developed a 3D-coculture model using primary ECs and SMCs derived from patients with AAA using poly-co-glycolide scaffolds that mimic the behavior of the medial aortic layer and exhibit mechanical properties and stiffness of AAA (112), thus serving as a promising model to understand the mechanobiology of AAA. To capture the dynamic stiffness and mechanosensing profile of SMCs, an ultrasound tweezers-based micromechanical system has been utilized to capture the mechanosensation of SMCs isolated from murine AAA (52). Transmission electron microscopy (TEM) and AFM can also be used to capture and map the structural and mechanical properties of viable human aortic SMCs (113, 114) and tissues. The recent advancement of the automatic patch-clamp system can also provide unlimited opportunities to directly measure the gating of ionic currents using an electrical force to induce cell stretching, thus allowing a recording in the membrane–matrix sites and the changes of physiological mechanosensors (98, 115, 116).

### 5.2. Clinical perspective: Monitoring disease progression and possible therapies

Multiple studies have shown that mechanical alterations within the AAA architecture can be utilized as a marker of the progressing phases of AAA, focusing on advanced imaging techniques and high-throughput computational methods. Previously, the changes in aortic pulse wave velocity (PWV), which allows the measurement of aortic stiffness, were reported as a promising imaging readout to monitor disease progression not only in mice but also for post-surgical prognosis of patients with AAA (117–119). However, the use of PWV to predict AAA risk needs to be evaluated in larger AAA cohorts in longitudinal studies in order to be able to be implemented clinically. Computational fluid dynamics (CFDs) is a widely used technique to investigate the potential risk of aneurysm rupture based on SS frequencies, blood flow, and changes in pressure (99, 120). However, CFD requires solid computational expertise to precisely and rigorously construct stimulations and perform numerical modeling (geometrical segmentation, fluid definition and domain) for each patient with AAA. Therefore, CFD is not yet clinically implemented and would necessitate concerted efforts from computational engineers to develop a platform using large-scale stimulations for the fast processing of geometry segmentation in a cost- and time-effective manner. Regardless, it was shown that maximum diameter and maximum wall stress were observed in the central aneurysm region, while regions with LSS (<0.4 Pa), larger curvature, and deposition of thrombus were associated with AAA expansion and higher risk of rupture (2, 3, 121). Using a CT angiography performed in 295 patients with AAA, LSS at baseline was associated with aortic expansion in a large clinical cohort, independent of any risk factors (3). It was also observed that 4D flow cardiovascular magnetic resonance images (MRIs) of the whole aorta were able to compute lower peak LSS in AAA compared to elderly controls and could be validated as a way to predict aneurysmal growth or rupture (4). Conversely, one retrospective clinical study reported greater aortic wall tension as a significant predictor of AAA rupture compared to the aortic diameter, even though this data has to be confirmed in larger prospective cohorts (122).

The Nobel-awarded study of David Julius and Ardem Patapoutian in 2021 (123) for the discovery of Piezo1 (51, 124) has further expanded its role in regulating vascular remodeling in AAA (52). Targeting Piezo1 is highly desirable as its expression peaks in established AAA, and its inhibition will likely break the cascade of signals responsible for promoting matrix degradation in AAA (52). Recently, small molecules of Piezo1 mediators have been generated and reported in the literature. Yoda1, an activator of Piezo1, has been shown to selectively activate Piezo1 but not Piezo2 (125). Conversely, spider toxin GsMTx4 was shown to suppress Piezo1 (126) effects. However, previous studies have reported off-targets of GsMTx4 such as Piezo2 and the TRP channel (127). There is, therefore, the necessity to generate more-selective and specific compounds to inhibit Piezo1.

Other mechanical targets such as AT1R blocker, Losartan and Telmisartan, were shown to slow AAA growth in hypertensive and Marfan syndrome mice (69, 72). However, none of these discoveries have yet to been reproduced in clinical trials (45). The selectivity of these blockers also remains an issue as an *in vivo* study performed in Marfan mice treated with calcium channel blockers targeted extracellular AT1R-mediated ERK1/2 activation and resulted in deleterious effect on aortic SMCs, therefore, accelerating aneurysmal growth in the ascending aorta (128).

The availability of compounds targeting ROS and inflammation is another attractive future target for AAA treatment. Alpha-ketoglutarate, a pleitropic antioxidant, has been shown to reduce ROS generation in C57BL mice challenged with pancreatic elastase (129). Exogenous antioxidants such as folic acid, vitamin C, and vitamin E have been reported to inhibit ROS production in several animal models such as ApoE^–/–^ mice and elastase-induced rat AAA models (130–132). However, no confirmatory studies have been performed in clinical subjects much needed to confirm the benefits of these agents in limiting AAA growth.

As the road to target mechanotransduction signaling pathways for therapeutic intervention remains an underdeveloped field, future works need to primarily address the exact contribution of key mechanosignaling pathways using available high-throughput systems that can replicate the microenvironment of the human aneurysm and precisely record the dynamic changes in both *in vitro* and *in vivo* models. The interrelated pathways between mechanosensors and other critical pathways in AAA may also hint that poly-pharmaceutical agents that can target both mechanosignaling and intrinsic cellular pathways are a more practical approach to prevent and limit AAA growth.

## 6. Concluding remarks

Mechanosensory and mechanotransduction machinery are typifying features inherent to AAA development. The formation of AAA is influenced by altered mechanical stress that amplifies pathological cellular signaling through the interaction between the stresses and mechanosensors present at the cell membrane of ECs and SMCs. In addition, increased ROS levels generally coincide with mechanotransduction during AAA, acting as critical second messengers that modulate several signaling pathways that participate in vascular remodeling, inflammation, and apoptosis. Further mapping of the mechano-behaviors in human AAA capable of capturing the dynamics of mechanical perturbation of the complex human aorta at critical phases of AAA development using sophisticated imaging and high-throughput technologies is still warranted. Further translational research is needed to test the relevance of the observations of defective mechanosensitive signals from murine to human disease. One critical step would be the use of large animal models such as porcine AAA models to test whether locally modulating mechanical signals during AAA onset would be of beneficial clinical interest. Importantly, strategies to intervene on these mechanical signals spatiotemporally are important factors to consider as most AAA are detected beyond their initial phase, bypassing an interventional window during the stages of AAA onset. Therefore, there is a dire need to discover novel druggable targets focused on altering mechanical signaling pathways during the progressing phases of AAA as the future of independent non-interventional therapies or in conjunction with current surgical approaches.

## Author contributions

BR and MP conceptually developed and supervised the project and co-wrote the manuscript. AR, CL, PK, and MP did the literature search and wrote the specific sections. CR, RH, and JV edited the text, provided the feedback, and discussion. All authors contributed to the article and approved the submitted version.
